# Advances on two serological assays for human papillomavirus provide insights on the reactivity of antibodies against a cross-neutralization epitope of the minor capsid protein L2

**DOI:** 10.3389/fimmu.2023.1272018

**Published:** 2023-11-08

**Authors:** Filipe Colaco Mariz, Kerstin Putzker, Peter Sehr, Martin Müller

**Affiliations:** ^1^ Tumorvirus-Specific Vaccination Strategies (F035), Deutsches Krebsforschungszentrum (DKFZ), Heidelberg, Germany; ^2^ EMBL-DKFZ Chemical Biology Core Facility, European Molecular Biology Laboratory, Heidelberg, Germany

**Keywords:** human papillomavirus, L2, neutralization assay, ELISA, vaccine, cross-neutralizing antibodies

## Abstract

**Introduction:**

A second generation of prophylactic human papillomavirus (HPV) vaccines based on the minor capsid protein L2 has entered clinical trials as promising alternative to meet the gaps left out by the current vaccines concerning type-restricted protection, high costs and low penetrance in immunization programs of lowand middle-income countries. Most of the serological assays available to assess anti-HPV humoral responses are, however, not well suited for measuring vaccine-induced anti-L2 antibody responses.

**Methods:**

In this work, we have advanced our automated, purely add-on High-Throughput Pseudovirion-Based Neutralization Assay (HT-PBNA) in an L2-oriented approach for measuring antibody-mediated neutralization of HPV types 6/16/18/31/33/52/58.

**Results and discussion:**

With the optimized settings, we observed 24- to 120-fold higher sensitivity for detection of neutralizing Ab to the L2 protein of HPV6, HPV16, HPV18, and HPV31, compared to the standard HT-PBNA. Alternatively, we have also developed a highly sensitive, cell-free, colorimetric L2-peptide capture ELISA for which the results were strongly concordant with those of the advanced neutralization assay, named HT-fc-PBNA. These two high-throughput scalable assays represent attractive approaches to determine antibody-based correlates of protection for the HPV L2 vaccines that are to come.

## 1 Introduction

The currently licensed HPV vaccines are greatly immunogenic and effective in preventing cervical intraepithelial neoplasia (CIN) and cancer (1, 2). Recent reports have also shown that all three vaccines – Cervarix, Gardasil and Gardasil9 – are able to induce long-lasting protection (>10 years after vaccination) against infection and pre-malignant lesions associated to HPV (3, 4). The HPV vaccines are based on recombinant, non-infectious virus-like particles (VLPs) formed by the L1 major capsid protein (5). Such particles are shown to present a morphology closely mimicking the HPV virions (in which L1 is arranged in 72 pentamers), triggering the stimulation of humoral immune responses with multiple copies of the virus dominant epitopes found on the surface loops of the viral capsid. The vaccines differ in their antigen composition, adjuvants formulation and the antigen expression system used for production. The establishment of worldwide HPV vaccination programs are, however, hampered by the high cost and complex supply-chain distribution of these cooling-chain dependent vaccines in developing countries, where nearly 90% of all fatalities by cervical cancer occur. According to current predictions, the number of cervical cancer cases is expected to rise, reaching up to 700,000 cases and 400,000 deaths by 2030 (6). The Global Strategy towards cervical cancer elimination recently adopted by the World Health Assembly defines as one of the goals to fully vaccinate 90% of the girls by the age of 15 (7), which is extremely challenging even in high-income settings (8, 9). Furthermore, the number of HPV-associated oropharyngeal cancer cases has been raising over the past two decades worldwide (reviewed by Shinomiya and Nibu (10)). In the United States between 1999 and 2015, the annual incidence of oropharyngeal cancer cases has increased 0.8% in women and 2.7% in males, the incidence increased by 2.7%, whereas cervical cancer incidence rates decreased 1.6% per year.

Neutralizing antibodies (nAbs) binding L1 loops are regarded as the primary mechanism of protection against HPV induced upon vaccination (11). Therefore, a number of functional assays have been developed to measure HPV nAb levels as correlate of vaccine efficacy. Pseudovirion-Based Neutralization Assays (PBNA) are considered the reference standard for measuring protective antibody levels to HPV, as it assesses a pertinent *in vitro* antibody activity. There are, however, a number of arguments making a case for the need to advance the HPV neutralization assays. Of pivotal importance is the fact that currently available PBNA is sub-optimal for detection of nAbs to the L2 minor capsid protein.

The L2 protein has been explored as alternative immunogen in a number of vaccine approaches. Differently from the VLPs, which induce a type-specific neutralizing antibody response targeting discontinuous epitopes displayed on the L1 loops (11, 12), the protection triggered by L2-based vaccines target continuous, highly conserved epitopes (13, 14) that can be presented out of the context of the HPV capsid making production in less complex, prokaryotic expression systems possible. Although not as immunogenic as the HPV VLP vaccines, the main attractiveness of L2-based experimental strategies rely, therefore, on their low-cost of production and broad spectrum of protection, since the most promising vaccine candidates were demonstrated to induce cross-neutralizing responses to several homologous and heterologous PVs (15). Detection of cross-neutralizing Abs (cnAbs) to L2 is, however, undermined in the current HPV PBNA settings, as demonstrated by Day et al. (16) when a vaccine candidate found to induce protective responses against cervico vaginal challenge, did not yield measurable nAb levels by the standard PBNA. These observations grounded the basis of further elegant methodological developments reunited in two PBNA variations: the so-called L2-PBNA (16) and the FC-PBNA developed by Roden and colleagues (17). In common, these assays enforce the L2 N-terminus exposure in the pseudovirion particles and allow increased sensitivity for L2 antibodies.

We have recently succeeded in establishing a high-throughput PBNA (HT-PBNA) for measuring nAb responses to several HPV types with great reproducibility and sensitivity (18–20). As the number of L2-based experimental vaccines currently approaching clinical studies is expected to rise (21–24), here we have envisioned the need to advance our HT-PBNA in order to achieve greater sensitivity to L2 nAbs of low- and high-risk HPVs. Alternatively, we also designed an enzymatic-linked immunosorbent assay (ELISA) based on peptides for measuring total anti-L2 antibody responses as a simpler, yet highly sensitive immunological method. We show that the peptide ELISA has good predictive value for the screening of broadly neutralizing antibody responses but, interestingly, the correlation between ELISA and PBNA varies among different HPV types.

## 2 Materials and methods

### 2.1 Antigen production and purification

The Pf Trx8merOVX313 vaccine candidate (25) used in this work was produced under a GMP-compliant protocol devised by Biomeva GmbH (Heidelberg, Germany). A research cell bank based on *Escherichia coli* BL21 strain was established and used for production of the recombinant antigen in a 10 liter fermenter following culture conditions established by the company. At the end of the fermentation process, the culture broth was subjected to lysis by two cycles of high-pressure homogenization at 950 bar. The resulting total lysate was heated to 65°C for 30 minutes to remove major fractions of host cell proteins and other constituents. Cell debris and precipitated impurities were removed by centrifugation and the pH of the final cleared lysate was adjusted to 8.

The conditioned product obtained after lysis and clearing steps was loaded to a capture column based on SP Sepharose FF (GE Healthcare), washed from impurities using 25 mM TRIS, 360 mM NaCl pH 8, and eluted with 25 mM TRIS, 600 mM NaCl pH 8. A further polishing step was conducted by applying the protein eluate from the capture column to Phenyl Sepharose HP equilibrated with 20 mM sodium phosphate, 1.5 M sodium chloride, 250 mM ammonium sulfate, 10 mM L-cysteine pH 6. After a washing step with 20mM sodium phosphate, 750 mM sodium chloride, 125 mM ammonium sulfate, 10 mM L-cysteine pH 6, the antigen was eluted with 20 mM sodium phosphate, 10 mM L-cysteine pH 6. The final antigen product was formulated in 1x PBS containing 2 mM L-cysteine and 0.03% Tween-20 and filtered over a 0.2 µm membrane.

### 2.2 Antibody samples, animal experiments and ethic declarations

#### 2.2.1 Mice sera

A group of ten, six- to eight-weeks old female BALB/c mice, purchased from Charles River Laboratories (Sulzfeld, Germany) and kept under specific-pathogen-free conditions (animal permit G248/16, Regierungspräsidium Karlsruhe, Germany), was immunized four times at biweekly intervals with 20µg of the Pf Trx8merOVX313 antigen (25), adjuvanted with 50% (v/v) AddaVax (InvivoGen) and administered in 50µl doses intramuscularly. Four weeks after the last immunization, animals were sacrificed and blood samples were collected by cardiac puncture. Following the sera retrieve, all samples were pooled into a single mix.

#### 2.2.2 Rabbit sera

Thirty-two 14-18 weeks old New Zealand White Crl: KBL(NZW) rabbits (equal number of males and females) were subjected to a local tolerance and repeated dose toxicity study performed at the Charles River Laboratories France Safety Assessment (standard project authorization no. 2017112118149040 approved by French authorities). Half of these animals were immunized four times at three-weeks intervals with 0.5mg of the Pf Trx8merOVX313 antigen adjuvanted with 0.5 mg aluminium hydroxide plus ALF-liposome (Army Liposome Formulation – Alpha, Walter Reed Army Institute of Research - WRAIR), whereas the other half received the adjuvant only. Blood samples were collected two days (d-66) and two weeks (d-79) after the last injection for serum retrieval. No abnormalities were observed during the evaluation of local reactions at the injection sites.

#### 2.2.3 Guinea pig sera

Eight outbread Hartley (Crl : HA) female guinea pigs (150- to 200g), obtained from Charles River Laboratories (animal permit A2/17, Regierungspräsidium Karlsruhe, Germany), were set in groups of two and immunized with 30 µg of four different PfTrx-L2-related antigens targeting up to nine cutaneous HPV types (23). All antigens were formulated in 50% (v/v) AddaVax and administered in 100 µl doses, four times at biweekly intervals, subcutaneously. Blood samples were collected by cardiac puncture four weeks after the last immunization.

#### 2.2.4 Human serum

Serum from a 21 years old female volunteer previously subjected to a 3-dose Gardasil schedule was collected and used by Sehr et al. (18) following obtainment of a written informed consent (ethics votum S-702/2021). The obtaining and testing of this sample was reviewed and approved by the Ethics committee University Heidelberg. This human serum was used as positive standard on all HT-fc-PBNA plates.

#### 2.2.5 Monoclonal antibodies

Two mice-derived monoclonal antibodies (mAbs) targeting the L2 20-38 amino acids of HPV16 and named K8 and K18 (13), were used as positive standard for L2-specific sensitivity.

### 2.3 HPV pseudovirion preparation

#### 2.3.1 Standard preparation

Pseudovirions (PSV) of HPV types 11, 16, 18, 31, 33, 52, and 58 were prepared by co-transfecting 293TT cells with plasmids encoding humanized L1 and L2 genes, and a reporter plasmid encoding the enzyme Gaussia-luciferase (GL). Briefly, 10µg of DNA were mixed to 30ug of polyethylenimine (PEI) in 1ml of unsupplemented DMEM and used to co-transfect 4x10^6^ 293TT in 10cm dishes. After three days of incubation at 37°C, 5% CO_2_, the transfected cells were harvested, washed in PBS (Invitrogen) and resuspended in 1ml of freshly-prepared lysis buffer containing 0.5% (w/v) Brij58 (Sigma) and 0.5% (v/v) RNase A/T1 mix (Thermo-Fisher) in DPBS (Invitrogen). This suspension was rotated for two days at 37°C to induce pseudovirion maturation. Then, a salt extraction procedure was employed with 0.17 volumes of 5M NaCl, followed by clearance of cell debris via centrifugation at 13.000 rpm for 15 minutes. The cleared lysate was then incubated in the presence of 250 units of benzonase (Turbo Nuclease, Jena Bioscience) for 1h at 37°C, and later subjected to purification by ultracentrifugation in Optiprep gradient. Fractions of the gradient were afterwards used to determine peaking PSV-derived reporter transduction in HeLa-T cells (18). Amounts of HPV6 PSV were not sufficiently active for the high-throughput assays.

#### 2.3.2 Furin-cleaved pseudovirion

An alternative protocol was used for preparation of eight furin-cleaved PSV types (fc-PSV, those already mentioned plus HPV6). In this case, co-transfections were carried out in furin-overexpressing 293TTF cells (17) and fc-PSV maturation conducted in the presence of 5mM CaCl_2_. The fc-PSV particles were purified as described above and tested in transduction assay with HeLa-T cells and furin-defficient LoVoT-cells (17), the latter one being selectively transducible only by fc-PSV particles.

### 2.4 Manually-conducted (FC-)PBNA

Serum samples were serially titrated (1:50 to 1:12,150) on 96-well plates containing DMEM (Sigma) supplemented with 10% FCS (Gibco), 1% penicillin/streptomycin (Life Technologies) and 200µg hygromycin B. All sera were tested in duplicates. Next, PSV diluted in DMEM (according to the dilution which yielded 10^6^ relative luminescence units (RLU) in the transduction assay) were added to the sera and the plates were incubated for 15 minutes at room temperature to allow binding of antibodies to the PSV. The assay preparation is finished with the addition of 12,500 HeLa-T cells per well and the plates are incubated at 37°C, 5% CO_2_ for two days. Detection of GL signal in the cell culture supernatant was assessed with the addition of coelenterazine (Gaussia Glow Juice kit, PJK), according to the manufacturer’s instructions. Neutralizing antibody titers (EC50) were calculated as the serum dilution which causes 50% inhibition of GL signal. Neutralization of fc-PSV were assessed with the use of furin- deficient LoVo-T cells (17) (formally known as FC-PBNA). For the serum or antibody samples displaying EC50 > 12,150 (the upper assay limit), neutralizing antibody titers were interpolated by GraphPad 8.3.1 using a dose-response – inhibition equation with variable slope. EC50 values <50 were considered non-neutralizing.

### 2.5 High-throughput pseudovirion-based neutralization assay

A detailed description of the HT-PBNA was done previously (18). Herein, different settings applied only to the serum dilution range (1:4 to 1:18,289) and number of LoVo-T cells employed (6000 cells/well). For the sake of clarity, the term HT-fc-PBNA was used when fc-PSV particles were incorporated into the assay, so to differentiate it from the use of regular, non-cleaved PSV particles. Assay cut-off was determined based on the mean EC50 value (100) measured in sera of non-immunized (placebo) rabbits. Serum or antibody preparations displaying EC50 ≥ 100 were considered neutralizing positive.

### 2.6 Cell viability assay

The effect of cell density on cell proliferation was tested using ATPlite 1step™ assay kit (Perkin Elmer). Using four 384-well plates, a total of 10000, 8000, 6000, 4000, 3000, 2000, 1500 and 1000 cells per well (in 16 wells per condition) of LoVo-T and HeLa-T were seeded in DMEM (Low Glucose with Pyruvate and L-Glutamine, Sigma) supplemented with 10% FCS (Gibco, BRL), 1% Pen/Streptomycin (Life Technologies), 1% L-Glutamine (Sigma) and 125µg/ml Hygromycin B (Roche). Cell density was monitored by counting the cells in a Neubauer chamber and in a Luna cell counter 24, 48, 72 and 96 hours after seeding. On these corresponding time points, cell viability was assessed by the quantification of luminescence signal upon addition of ATP, luciferase and D-luciferin following the manufacturer’s recommendation. Luminescence reporter signal is directly correlated with the cell number/metabolic activity. Cell confluency (%) and viability (in RLU) are represented as the average values measured in 16 wells.

### 2.7 Enzyme-linked immunosorbent assay using HPV L2-peptides

Serocluster 96-well “U” bottom plates (Costar, USA) coated overnight at 37°C with 0.2 µg/well of streptavidin (Sigma-Aldrich, Germany) in PBS and blocked for 1h at room temperature with PBS buffer containing 1.5% skim milk and 0.3% Tween, were used to capture N-terminally biotinylated HPV L2 peptides (corresponding to the GGSG linker followed by an L2 aa region homologous to the HPV16 L2 20-38 aa epitope, GenScript Biotech, Netherlands) diluted to 0.1 µg/well in blocking solution. The capture of L2 peptides was performed for 1h at room temperature and unbound peptides were washed away with PBS containing 0.3% Tween. Then, samples were serially diluted (in duplicate) in the plate using blocking solution in 3-fold steps (ranging from 1:1,050 to 1:255,150 for the polyclonal sera and from 1:50 to 1:12,150 for the monoclonal antibody preparations). Plates were incubated for 1h at 37°C to allow antibody binding to the L2 peptides, followed by another washing step to remove unbound antibodies. Next, HRP-conjugated goat anti-rabbit or -mouse IgG (Southern Biotech, USA) diluted 1:3,000 in blocking solution was added to the plates for another 1h at 37°C. Following another washing step, the presence of anti-L2 antibodies was revealed by adding 100 μl/well of 2,2′-azino-bis(3-ethylbenz-thiazoline-6-sulfonic acid) (ABTS; 1 mg/ml in 100 mM sodium acetate-phosphate buffer, pH 4.2, containing 0.015% H_2_O_2_) and measuring the optic density at 405 nm with a Multiskan Go (Thermo Fisher Scientific, USA) after 15 minutes. Anti-L2 antibody titers (IC50) were determined as the sample dilution leading to an average absorbance corresponding to 50% of the assay saturation (highest absorbance value) for each peptide. Background reactivity was defined as the average absorbance measured across the sera of unvaccinated rabbits (placebo).

### 2.8 5,5-di-thiobis-(2-nitrobenzoic acid) assay

The Ellman’s reagent, 5,5-di-thiobis-(2-nitrobenzoic acid) or DTNB, was used to determine the amounts of free sulfhydryl (-SH) groups and consequent reduced cysteine in the L2 peptide suspensions. The assay was performed with 15 mM peptides in 200 µl volume containing 40 mM DTNB (GoldBio, USA), 100 mM sodium phosphate, 1 mM EDTA, pH 8. A serial dilution of N-acetyl-cysteine (Sigma-Aldrich) was used to build a standard curve, and the concentration of 5-thio-2-nitrobenzoic acid (TNB) molecules formed upon reduction of DTNB disulfide bounds was measured with an optical density at 412 nm (according to the formula CSulfhydryl = OD412 - Yint of Std. Curve/Slope of Std. Curve) in a Multiskan Go (Thermo Fisher Scientific, USA). The percentage of reduced cysteine was estimated by dividing the number of DTNB-reactive –SH groups by the amount of peptide used for the assay.

### 2.9 Statistics

HPV mean neutralizing and total anti-L2 antibody titers (EC50 and IC50, respectively) with standard error of mean, and nonparametric Spearman rank correlation coefficients (r) with corresponding 95% confidence intervals (using the method of Clopper-Pearson) were calculated using GraphPad 8.3.1. Statistical significance (two-tailed) was determined with the nonparametric Mann-Whitney test, also via GraphPad.

## 3 Results

Incorporation of fc-PSV into the HT-PBNA increases sensitivity to L2 neutralizing antibodies

The use of HPV fc-PSV in neutralization assay has been successfully demonstrated by others (17, 26), but considerable variations were applied to our protocol. Importantly, we employ a reporter system based on HeLa-T cells and the GL gene (18). Using a 96-well plate format and manual set-up, we firstly assessed the nAb titers to HPV types 16, 18 and 31 in a pool of sera obtained from 10 mice vaccinated with the Pf Trx8merOVX313 (25) (PANHPVAX, an L2-based vaccine targeting seven high-risk HPV types, plus HPV6), and a guinea pig serum raised against another L2-based vaccine candidate targeting nine cutaneous HPV types (the Pf Trxc9mer) (23).

**Figure 1:**
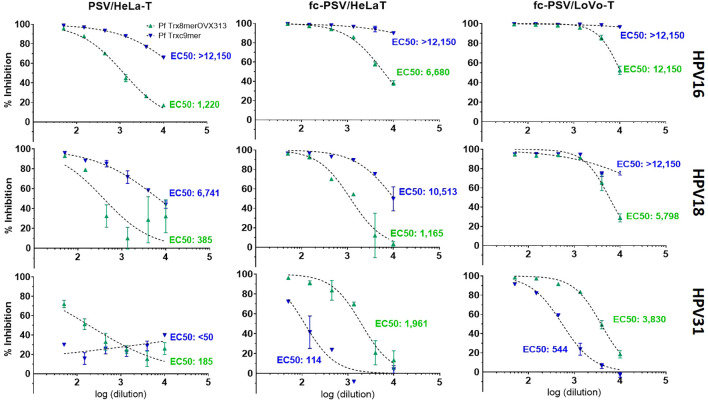
Comparison of neutralizing antibody titers to HPV types 16, 18 and 31 measured by PBNA with PSV, fc-PSV, HeLa-T and LoVo-T. Each chart shows pseudovirion inhibition (%) obtained upon mixing PSV or fc-PSV particles with a serial dilution (log_10_) of anti-L2 immune sera (green – anti-Pf Trx8merOVX313; blue anti-Pf Trxc9mer). Each data point (triangle) corresponds to the average inhibition obtained from duplicates with vertical bars displaying the 95% confidence intervals. The EC50 values represent the neutralizing antibody titers yielding 50% PSV inhibition. For the serum samples displaying EC50 > 12,150 (the upper assay limit), neutralizing antibody titers were interpolated using a dose-response – inhibition equation with variable slope. EC50 values <50 were considered non-neutralizing.

With the use of fc-PSV and HeLa-T (**Figure 1**), we observed a 5.4-fold higher nAb titer to HPV16 for the pooled sera (EC50 of 6,680), as compared to the standard PBNA method using non-cleaved PSV (EC50 of 1,220). Using LoVo-T, a furin-deficient cell line demonstrated to be selectively infected by fc-PSV only, the increase was 10-fold (EC50 of 12,150). Similarly, we observed 3- and 15-fold increased L2 nAbs titers in the pooled sera to HPV18 with the use of fc-PSV and HeLa-T (EC50 of 1,165) or LoVo-T reporter cell line (EC50 of 5,798), respectively, as compared to the standard PBNA (EC50 of 385). For HPV31, we found 10- and 20-fold increased L2 nAb titers in the pooled sera with the fc-PSV and HeLa-T (EC50 of 1,961) and LoVo-T (EC50 of 3,830), respectively, as compared to the standard PBNA (EC50 of 185).

For the guinea pig serum, the calculated incremental sensitivity afforded by the use of fc-PSV was based on interpolated EC50 values obtained with a dose-response - inhibition equation with variable slope. Notably, we found consistently stronger antibody levels in the combination fc-PSV and LoVo-T cells as compared to fc-PSV and HeLa-T cells. For instance, the use of HPV16 PSV and HeLa-T yielded nAbs titers of 26,900 compared to 505,630 measured by HPV16 fc-PSV and HeLa-T cells. Instead, 26-fold higher nAbs titers were found with HPV16 fc-PSV and LoVo-T cells (EC50 of 15,106,450) as compared to those measured with the same fc-PSV but using HeLa-T). The use of HPV18 PSV and fc-PSV with HeLa-T also led to EC50 of 6,741 and 10,513, respectively for the same serum. Combining fc-PSV and LoVo-T cells, 9-fold higher nAb titers were obtained (EC50 of 96,690) against HPV18. For HPV31, L2 nAb levels were not detected for the guinea pig serum with the use of non-cleaved PSV, whereas 3.7-fold higher titers were found with the use of fc-PSV in LoVo-T (EC50 of 544) than in HeLa-T (EC50 of 114). These observations encouraged us to assess the use of fc-PSV and LoVo-T cells in our HT-PBNA setting.

We observed 24- to 120-fold higher L2 nAb levels to HPV6, HPV16, HPV18 and HPV31 in the pooled sera of mice vaccinated with the Pf Trx8merOVX313 antigen, by incorporating fc-PSV instead of standard (i.e. non-cleaved) PSV in the high-throughput setting (HT-fc-PBNA/HeLa-T; **Table 1**). A 127- to 350-fold increase in nAb levels to HPV16, HPV18 and HPV31 was observed upon employment of LoVo-T and fc-PSV in the high-throughput setting, as compared to the standard HT-PBNA (with HeLa-T and non-cleaved PSV, **Table 1**). Increased nAb levels to the L1 protein of HPV6, HPV16 and HPV18 were also seen with the HT-fc-PBNA/LoVo-T setting, although these observations derived from only one human serum (**Table 1**).

**Table 1:** Neutralizing antibody levels measured in mice¹ and human² sera by the HT-PBNA and HT-fc-PBNA using 384-well plates.

Mice anti-L2 serum pool (EC50)				Human Gardasil anti-L1 serum (EC50)		
	HeLa-T		LoVo-T	HeLa-T		LoVo-T
	PSV	fc-PSV	fc-PSV	PSV	fc-PSV	fc-PSV
**HPV6**	36	4,762	4,720	17,106	132,022	>180,000
**HPV16**	129	3,130	45,566	13,325	22,668	62,223
**HPV18**	136	3,291	17,358	1,708	4,635	16,793
**HPV31**	<25	1,519	15,043	186	244	208

The use of fc-PSV and LoVo-T provide considerably higher sensitivity for detection of HPV types 6/16/18/31 L2 neutralizing antibody levels.

¹A pool of sera collected from 10 mice immunized with the Pf Trx8merOVX313 antigen.

²A human serum collected after immunization with Gardasil.

**Figure 2:**
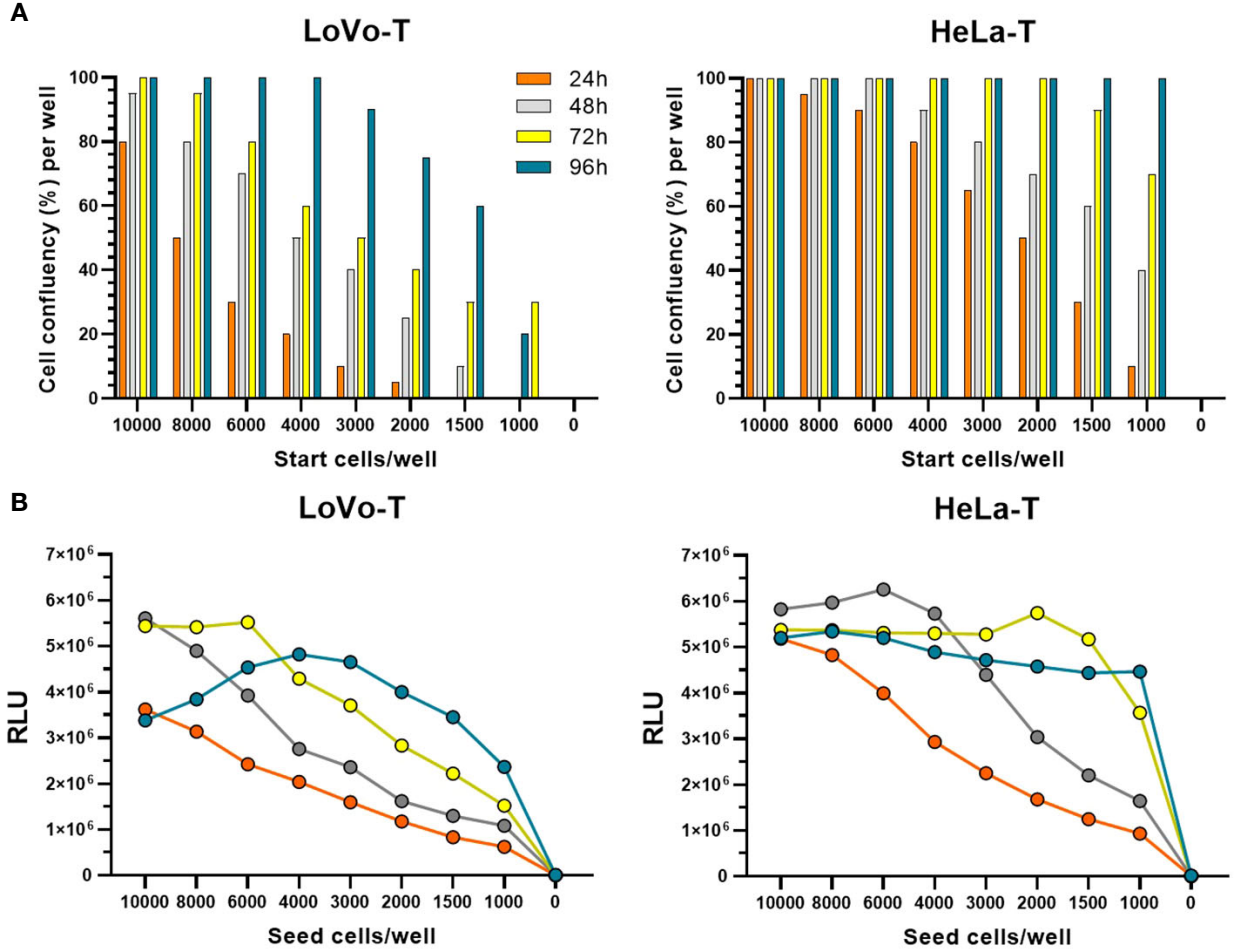
Comparison of HeLa-T and LoVo-T growth kinetics in 384-well plate format. **(A)** Cell confluency was estimated by microscopy. The legend indicates the time points when measurements were taken. **(B)** ATP is a marker for cell viability due to its presence in all metabolically active cells. The cell number was measured based on the production of light caused by the reaction of ATP with added luciferase and D-luciferin using the ATPlite 1step™ kit from PerkinElmer. The average of luminescence measured in 16 wells is shown.

As highlighted previously (18), the robustness of the PBNA towards variations in cell number and reporter signal is a crucial parameter for performing a high-throughput, automated assay. Therefore, we proceeded to evaluate in more detail the use of LoVo-T cells in the 384-well format, add-on assay layout devised for the HT-PBNA. In comparison to the HeLa-T cell line, the use of LoVo-T was associated to markedly differences in regard to growth behavior and GL reporter activity. We observed the need to employ four times more LoVo-T cells (6,000 cells/well) in order to reach similar cell density (60% confluency) as HeLa-T cell line (1,500 cells/well) in the course of 48h of growth (**Figure 2A**). Determination of ATP levels over days of cell growth, a method used for inferring cell number and viability, also showed that HeLa-T cells are able to produce 2-fold higher signal as compared to the LoVo-T cell line (**Figure 2B**), providing additional evidence of distinct growth behavior for the two report cell lines. Additionally, when the transduction ability was assessed with a HPV16 fc-PSV, we found 1/20th (median of 3.9E+06 RLU) and five-fold more variable (CV of 20%) reporter signal with LoVo-T cells, as compared to HeLa-T cells (median of 9.0E+07 RLU; CV of 4.3%) (**Table 2**). Further drawbacks related to the use of LoVo-T cells in the HT-PBNA included a less efficient, not uniform trypsinization treatment and delayed cell attachment, in comparison to HeLa-T (data now shown). For these reasons, we decided to use Hela-T cells in the HT-PBNA, despite the reduced sensitivity compared to LoVo-T cells in detecting anti-L2 neutralizing antibodies, as discussed above.

**Table 2:** HPV16 fc-PSV-driven transduction efficiency of HeLa-T and LoVo-T reporter cell lines determined in 384-well plate after addition of coelenterazine.

Gaussia-luciferase activity (RLU)		
	HeLa-T	LoVo-T
**Median**	9.0E+07	3.9E+06
**SD**	3.8E+06	0.8E+07
**Min**	7.9E+07	2.5E+06
**Max**	9.6E+07	7.3E+06
**% CV**	4.3%	20%

Gaussia-luciferase activity is reported in median relative luminescence units (RLU), with standard deviation (SD), minimal (min) and maximum (Max) values, and the corresponding coefficient of variation (CV) in percentage.

### 3.1 Increased sensitivity for detection of vaccine-induced anti-L2 neutralizing antibodies with the use of the adapted HT-fc-PBNA

Despite the high sensitivity of the HT-fc-PBNA with LoVo-T, this reporter cell line appeared to be ill-suited for the high-throughput setting. In all subsequent assays, we validated the HT-fc-PBNA/HeLa-T setting for detection of L2 nAbs in a panel of polyclonal and monoclonal antibodies.

**Figure 3:**
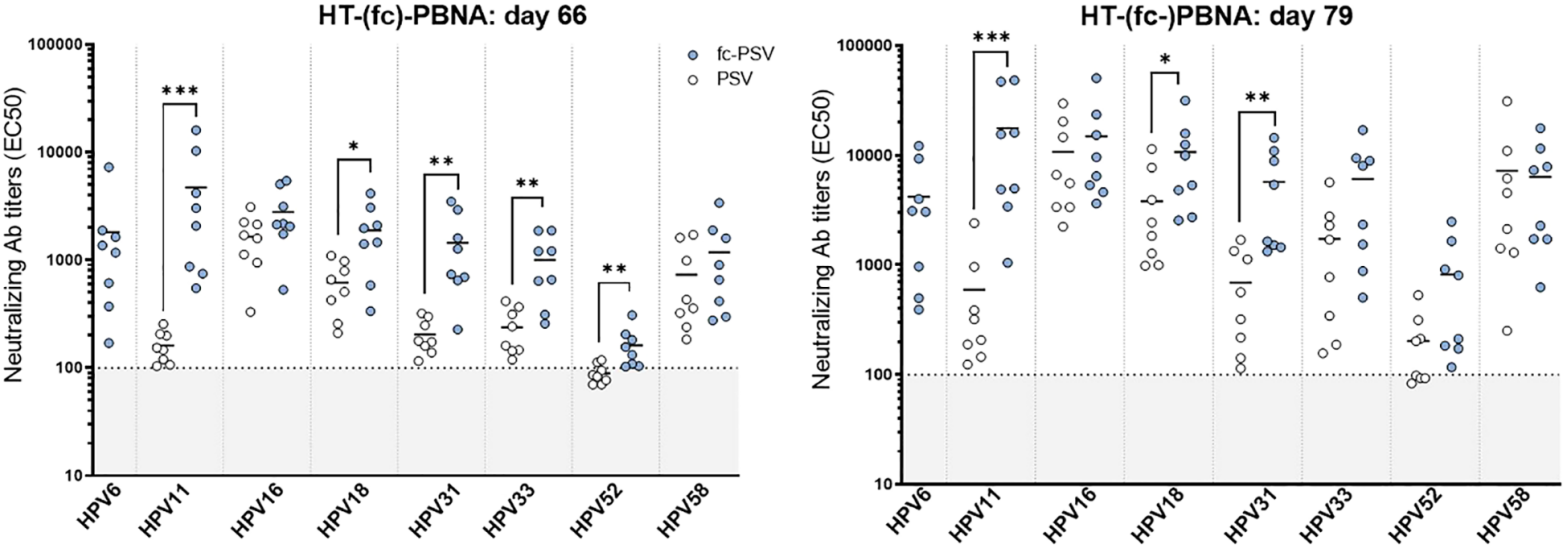
Detection of neutralizing antibody titers to eight mucosal HPV types in rabbit sera by the HT-(fc)-PBNA. Each data point corresponds to the neutralizing antibody titer (EC50) for one serum for a given HPV type. In blue dots, the EC50 values obtained with fc-PSV, whereas white dots represent the EC50 values calculated with non-cleaved PSV. Dashed lines denote the assay cut-off determined by the mean EC50 value measured in the rabbit sera of placebo group. A P-value of ≤0.05 was considered significant. *P < 0.05; **P < 0.01; ***P < 0.001, as determined by the nonparametric Mann–Whitney test.

Following a toxicology study with the Pf Trx8merOVX313 vaccine in 32 rabbits, we characterized the L2 nAb levels to HPV6/11/16/18/31/33/52/58 in sera collected at days 66 and 79 post-vaccination (16 samples per time-point). Except for HPV6 (which was tested only with fc-PSV), a direct comparison among Ab levels determined by the HT-fc-PBNA and the HT-PBNA was carried out in a single run. We observed significantly higher nAb levels to HPV11/18/31/33/52 at day 66 post-vaccination in the HT-fc-PBNA, as compared to nAb levels obtained in the HT-PBNA (**Figure 3**). In fact, mean nAb levels detected with the HT-PBNA were below or close to the cut-off value (EC50 of 100) for HPV11 (mean EC50 of 150), HPV31 (mean EC50 of 200), HPV33 (mean EC50 of 230) and HPV52 (mean EC50 of 90). These titers increased to 4,500, 1,345, 986 and 163, respectively, when measured by the HT-fc-PBNA. Mean nAb levels to HPV16 (mean EC50 of 1,618), HPV18 (mean EC50 of 600) and HPV58 (mean EC50 of 724) were 3- to 9-fold higher than the cut-off value and these increased, respectively, to 2,740, 1,830 and 1,150. The increased sensitivity observed in the HT-fc-PBNA was consistently observed also for samples collected 79 days post vaccination: 3- to 40-fold higher mean nAb titers to HPV11 (mean EC50 of 17,000), HPV18 (mean EC50 of 10,365) HPV31 (mean EC50 of 5,756), HPV33 (mean EC50 of 5,938) and HPV52 (mean EC50 of 820) in the HT-fc-PBNA, as compared to the HT-PBNA. For HPV6, no comparison was possible between the two assays, as it was only tested by the HT-fc-PBNA, yet, high mean nAb levels (EC50 of 4,095) measured for sera collected on day 79 were 22-fold higher than the assay cut-off.

**Figure 4:**
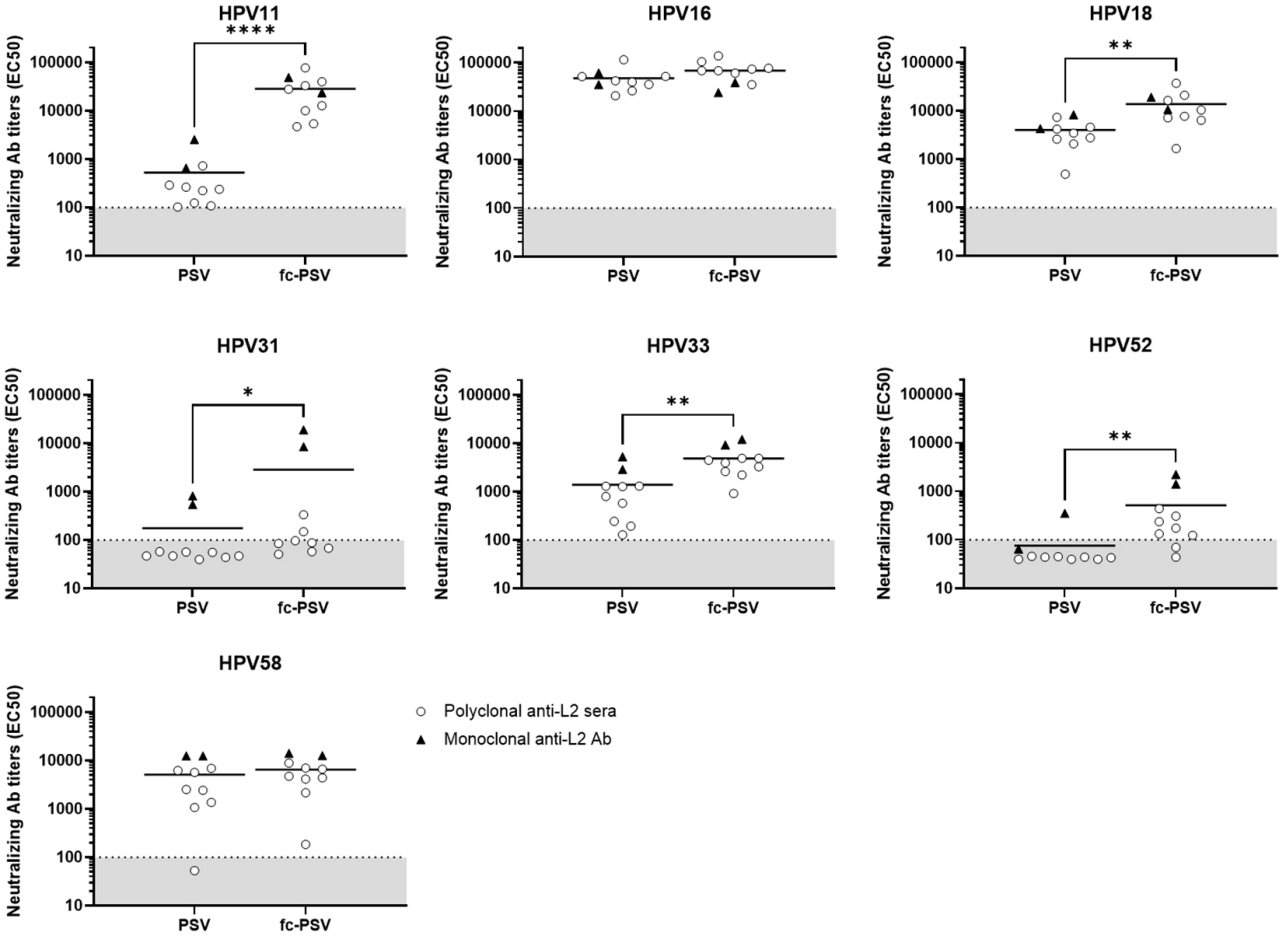
Detection of neutralizing antibody titers to eight mucosal HPV types in guinea pig sera and monoclonal antibody preparations by the HT-(fc)-PBNA. Each data point corresponds to the neutralizing antibody titer (EC50) calculated in one serum (white dot) or antibody preparation (black triangle) for a given HPV type. Horizontal bars represent the mean EC50 values calculated for all samples when tested by PSV or fc-PSV. Dashed lines denote the assay cut-off determined by the mean EC50 value measured in the rabbit sera of placebo group. A P-value of ≤0.05 was considered significant. *P < 0.05; **P < 0.01; ****P < 0.0001, as determined by the nonparametric Mann–Whitney test.

We also investigated the performance of the HT-fc-PBNA for characterization of cross-neutralizing anti-L2 antibodies in sera obtained from eight guinea pigs immunized with four different L2-based vaccine candidates against cutaneous HPV (23). Significantly higher mean nAbs levels to HPV11/18/31/33/52 were found when the HT-fc-PBNA was deployed, in comparison to the HT-PBNA (**Figure 4**). The gain in sensitivity varied from 3-fold for HPV18 and HPV33, 6-fold for HPV52, 16-fold for HPV31, to 53-fold for HPV11 (**Supplementary Table 1**). Mean nAb levels to HPV16 and HPV58 in the immunized guinea pigs were also higher in the HT-fc-PBNA, as compared to the HT-PBNA, but the differences were not statistically significant (**Figure 4**). Supporting the high sensitivity for the L2 nAbs of other HPV types observed in rabbit sera, we found cross-neutralizing Ab levels to HPV6 in all guinea pig sera, with the mean Ab levels being 77-fold higher than the assay cut-off value (**Supplementary Table 1**). Interestingly, the sensitivity pattern observed with the anti-L2 monoclonal antibodies (mAb) K8 and K18 followed the same trend seen for the animal sera. Previously shown to be cross-neutralizing (13), those mAb demonstrated increased reactivity to HPV11, HPV18, HPV31, HPV33 and HPV52 fc-PSV, compared to their standard PSV counterpart. On the other hand, no differences were seen for HPV58, regardless of the virus vector (PSV or fc-PSV). For HPV16 (to which they were originally generated) antibody titers were already very high when standard PSV was used, and no substantial increment on sensitivity was observed by applying fc-PSV.

### 3.2 Binding of anti-L2 antibodies to the peptides as a predictor for broad neutralizing ability

Although considered the gold-standard for measuring antibody-mediated neutralization responses, the HT-PBNA demands considerable technical challenges to be conducted. Thus, we devised a cell-free, colorimetric-based capture ELISA to detect antibodies against the L2 20-38 aa epitopes of different HPV types. With the use of streptavidin and biotinylated peptides comprising the L2 20-38 aa region, we observed good overall assay performance (i.e. low intra-assay variability and good sensitivity, **Table 3** and **Supplementary Figure 1**) for detection of anti-L2 antibodies.

**Table 3:** Limit of detection as determined for two anti-HPV16 L2 monoclonal antibodies (mAb) tested in the HT-PBNA, HT-fc-PBNA and L2-peptide ELISA according to the reported EC50 and IC50 values.

	Limit of detection (pmol/µl)								
Assay	HPV type								mAb
	6	11	16	18	31	33	52	58	
HT-PBNA	NA	6.6E-4	6.6E-8	2.6E-4	3.3E-3	4.6E-4	4E-2	2E-4	K18
HT-fc-PBNA	1.3E-4	5.3E-5	6.6E-8	1.3E-4	1.3E-4	2E-4	1.3E-3	2E-4	
L2-peptide ELISA	1.3E-3	1.3E-3	6.6E-4	6.6E-4	1.3E-3	2.6E-3	4.6E-3*		
HT-PBNA	NA	1.3E-3	1.3E-5	1.3E-4	1.3E-3	2.6E-4	Neg	6.6E-8	K8
HT-fc-PBNA	3.3E-5	3.3E-5	3.3E-5	6.6E-8	6.6E-8	6.6E-8	5.3E-4	6.6E-8	
L2-peptide ELISA	Neg	Neg	3.3E-4	Neg	1.3E-3	1.3E-3	3.3E-3*		

*L2-peptide ELISA IC50 value determined with the same HPV52/58 peptide.

Neg, negative.

**Figure 5:**
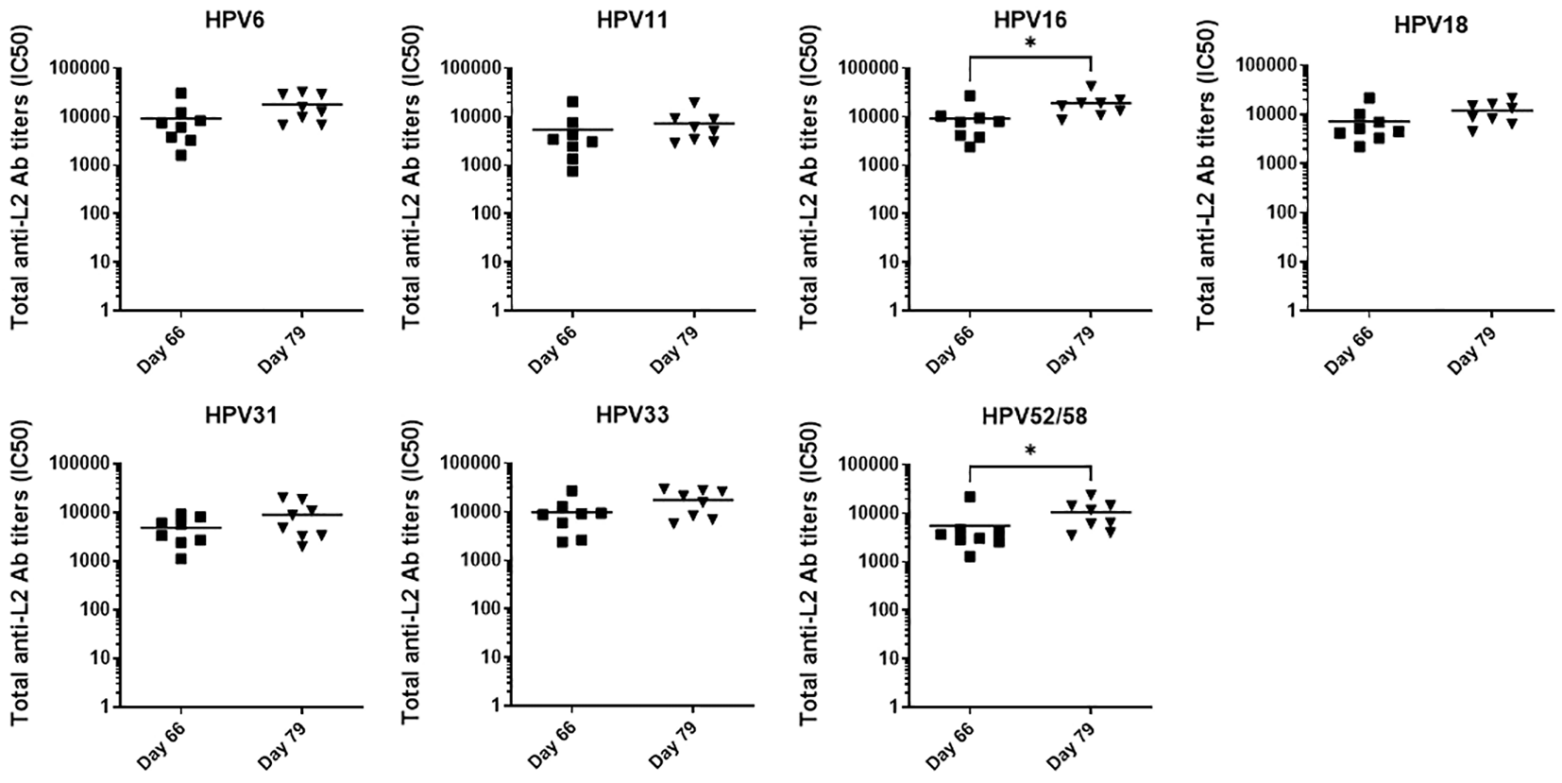
Detection of total anti-L2 antibody titers to eight mucosal HPV types by the L2-peptide ELISA. Antibody titers were compared in rabbit sera collected at days 66 and 79 post-vaccination. Each data point corresponds to the total anti-L2 antibody titer (IC50) calculated in one serum for a given HPV type. A P-value of ≤0.05 was considered significant. *P < 0.05, as determined by the nonparametric Mann–Whitney test.

We found anti-L2 total antibody titers to HPV types 6, 11, 16, 18, 31, 33, 52 and 58 in the rabbit sera at days 66 and 79 post-vaccination measured by the L2-peptide ELISA (**Figure 5**). Anti-L2 antibody titers were high for all types already in sera collected at day 66, with mean titers varying from 4,600 (HPV31) to 9,700 (HPV33). On day 79, we measured up to 2-fold higher median antibody titers than at day 66 for all types, and differences were statistically significant for HPV16, HPV52 and HPV58 (**Figure 5**). Therefore, the Ab titers determined by L2-peptide ELISA are in line with the higher sensitivity of the HT-fc-PBNA (**Figure 3**), and are markedly distinct from the overall weaker nAb titers measured for HPV52 by the HT-PBNA.

**Figure 6:**
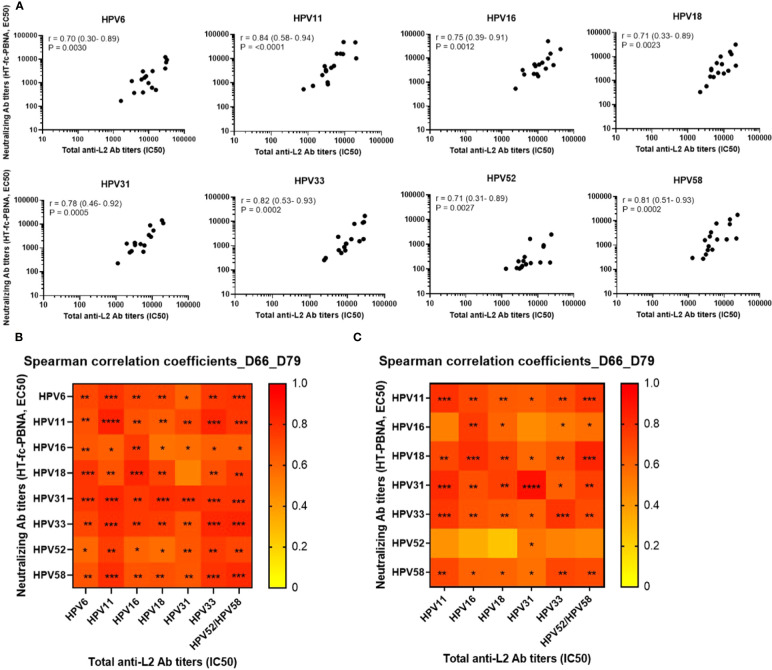
Correlation of neutralizing (HT-(fc)-PBNA) and total antibody titers to the L2 protein of HPV types 6, 11, 16, 18, 31, 33, 52 and 58. **(A)** Neutralizing antibody titers measured at days 66 and 79 by HT-fc-PBNA were correlated to the total anti-L2 antibody titers determined by L2-peptide ELISA for each HPV type. Total anti-L2 antibody titers across the eight HPV types were also correlated to the neutralizing antibody titers determined by the HT-fc-PBNA **(B)** and HT-PBNA **(C)**. r – Spearman rank correlation coefficients with 95% confidence intervals in parenthesis. A P-value of ≤0.05 was considered significant. *P < 0.05; **P < 0.01; ***P < 0.001, as determined by the nonparametric Mann–Whitney test.

The total anti-L2 antibody titers measured by the L2-peptide ELISA correlate with the nAb titers determined by the HT-fc-PBNA. Moderate to high positive Spearman rank correlation coefficients (r > 0.7) of total HPV type-specific anti-L2 antibody titers and nAb titers were observed, indicating an overall strong correlation between the L2-peptide ELISA and the HT-fc-PBNA results (**Figure 6A**). The strongest correlations were found for HPV11 (r = 0.8), HPV33 (r = 0.8) and HPV58 (r = 0.8) antibody titers. Moreover, we attempted to correlate the neutralizing and total anti-L2 antibody titers across different HPV types, given the high degree of sequence homology of the 20-38 aa epitope explored in our vaccine concept. Interestingly, we observed that total anti-L2 antibody titers measured for HPV types 11, 33, 52 and 58 are strongly and significantly correlated with HT-fc-PBNA neutralizing antibody titers for HPV6/11/16/18/31/33/52/58 (**Figure 6B**), and predominantly weaker correlations were observed for the HT-PBNA (**Figure 6C**).

Following on the indications that HPV11, 33, and 52/58 L2 peptides can be used to detect cross-reactive anti-L2 antibodies targeting the 20-38 aa regions and exhibiting broadly neutralizing ability, we determined the oxidization state of all peptides. In this regard, we observed that the oxidation state of the two conserved cysteine residues present in the L2 peptides varied substantially (**Table 4**): the HPV52/58 L2 peptide presented the highest oxidation state (90%), followed by the HPV33 (65%), HPV18 (65%), HPV31 (60%), HPV11 (59%), HPV6 (42%) and HPV16 (40%) L2 peptides. These findings, however, were not linked to strikingly different reactivity patterns, suggesting that an oxidation threshold of 40% is sufficient to provide type-specific peptide reactivity to antibodies with neutralizing activity. In line with this argument, we obtained essentially similar Spearman correlation coefficient of neutralizing antibody titers and total antibody titers for HPV18/33/58 (65-90% oxidized peptides, r = 0.7) and for HPV6/11/16 (40-60% oxidized peptides, r = 0.6) (**Table 4** and **Supplementary Figure 2**).

**Table 4:** Oxidation state (%) of the conserved cysteine residues present in the L2 peptides harbouring the aa 20-38 as determined by DTNB assay.

L2 peptides	Oxidation state (%)	r
HPV6 GGSGQTCKLTGTCPPDVIPKVEHN	42	0.7
HPV11 GGSGQTCKATGTCPPDVIPKVEHT	60	0.8
HPV16 GGSGKTCKQAGTCPPDIIPKVEGK	40	0.7
HPV18 GGSGKTCKQSGTCPPDVVPKVEGT	65	0.7
HPV31 GGSGQTCKAAGTCPSDVIPKIEHT	60	0.8
HPV33 GGSGQTCKATGTCPPDVIPKVEGS	65	0.8
HPV52/58 GGSGQTCKASGTCPPDVIPKVEGT	90	0.7/0.8

Corresponding Spearman correlation coefficients (r) calculated for type-specific L2-peptide ELISA and the HT-fc-PBNA assays are also provided.

## 4 Discussion

Neutralizing antibodies targeting the HPV major capsid protein L1 have been demonstrated to be protective against papillomavirus infection in different animal models (27–29). In humans, vaccine efficacy against HPV-related persistent infection and CIN lesions is well correlated to neutralizing antibody levels induced upon immunization with L1-based vaccines (3, 4, 30). Nevertheless, protective antibody levels are still not formally defined. Antibodies against the minor capsid protein L2 were also demonstrated to protect animals against experimental papillomavirus infection (21, 22), but the development of novel L2-based prophylactic vaccines against HPV was hampered in the past due to its lower immunogenicity compared to the L1-based VLP vaccines, despite of an attractive potential to trigger broadly protective responses demonstrated in pre-clinical studies. An important caveat of most past studies was the use of the first generation neutralization assays that were sub-optimal for measuring L2-specific neutralizing antibodies (15).

Studies led by P. Day insightfully demonstrated that cross-neutralization epitopes found in the amino-terminal portion of L2 are buried within the HPV capsid, consequently being restricted to recognition by antibodies (reviewed by Olczac & Roden (15)). In the course of early events of infection that take place at the basal membrane of the epithelium, when a series of conformational change in the capsid lead to the proteolytic cleavage by the furin convertase, the N-terminus region of the L2 protein becomes exposed and accessible for antibody binding, which affords virus neutralization and opsonization. Importantly, these molecular events are not mimicked adequately in the standard HPV PBNA, but were incorporated into the adapted FC-PBNA by R. Roden’s group (26). The translational success of L2-based vaccine candidates depends, therefore, on highly sensitive and straightforward immunological assays adapted for L2-specific antibodies.

In the context of our L2-based, polytope vaccine concept, we have already demonstrated that the aa region 20-38 induces high neutralizing antibody levels that not only confer protection against virus challenge with a broad array of cutaneous (23) and mucosal HPV types (25, 31), but also afford protective immunity against papillomavirus-related experimental infection and tumor development (22). With our lead vaccine candidate, PANHPVAX, recently entered into a phase-I clinical trial (NCT05208710), here we have advanced two immunological assays that are well-suited for detecting anti-L2 antibody responses with different but complementary characteristics. On the one hand, the HT-fc-PBNA demonstrated considerably higher sensitivity than its counterpart HT-PBNA for detection of L2 cross-neutralizing antibodies to eight mucosal HPV types, while keeping its original strengths, i.e. high-throughput, semi-automated layout. On the other hand, the simpler L2-peptide ELISA presented high sensitivity to detect total L2-specific antibody responses that greatly correlated with the HT-fc-PBNA data. Specifically, high total and neutralizing L2 antibody levels were measured by L2-peptide ELISA and HT-fc-PBNA for HPV types 11, 31 and 52, whereas HT-PBNA suggested rather weak or absent antibody responses.

Correlation of total anti-L2 antibody levels determined by the L2-peptide ELISA and broadly neutralizing activity (measured by HT-fc-PBNA) are strong, and the demonstration that the two cysteine residues presented within the 20-38 region of the peptides are oxidized might provide insights on the structural features of the cross-neutralization epitope within the aa region 20-38. This is in line with the demonstration that L2 residues Cys22 and Cys28 are disulfide-bounded in HPV16 virions (32), and led us to speculate that such intramolecular bond create a spatial configuration, likely a loop structure, that is required for recognition by anti-L2 neutralization antibodies. Such structures are inaccessible or sub-optimally presented in non-cleaved PSV particles and poorly oxidized L2 peptides, but become better displayed in furin-cleaved PSV particles (16, 17) and highly oxidized peptides. Because the Cys22 and Cys 28 residues are highly conserved among homologous and heterologous papillomaviruses, the highly-oxidized HPV11/33/52/58 L2 peptides presented an attractive reactivity pattern for screening of antibodies with broad neutralizing ability (against several HPV types). Interestingly, correlation of type-specific total antibody levels and neutralizing antibody levels to HPV6 and HPV16 were still strong despite the lower oxidation state of the corresponding peptide (42% and 40%, respectively). We think that this apparent ambiguity arise from the role of the primary peptide structure, additionally to the oxidation state of the cysteine residues, in determining the spatial configuration to which cross-reactive anti-L2 antibodies are bound. Importantly, we have shown that the corresponding cysteine residues are disulfide bonded when inserted into our thioredoxin-based scaffold, which contributes to the vaccine immunogenicity (33).

The main limitation of our study is related to the unfeasibility to incorporate LoVo-T cells into our HT-fc-PBNA reporter system. In our opinion, the use of LoVo-T cells would have allowed us to measure an even stronger vaccine immunogenicity (increased nAb levels), because these cells are selectively transducible by fc-PSV only (17). The nAb levels measured with the fc-PSV and HeLa-T in our study are probably masked (underestimated) by the presence of non-cleaved PSV particles also present in the fc-PSV preparation, that escape neutralization by the L2 antibodies and, therefore, drive transduction of reporter signal. Nevertheless, the combination fc-PSV/HeLa-T allowed substantially increased sensitivity as compared to the use of PSV particles, with higher robustness in our high-throughput settings. A minor limitation is related to the lack of comparative data for the HPV6 HT-PBNA. Although not considered a limitation, we would like to point out that PANHPVAX was adjuvanted with ALFA for the toxicology study in animals, which is not the one adjuvant (cyclic di-AMP) in use for the ongoing clinical trial. In our opinion, this observation does not affect the main conclusions of our study, which scope is to advance immunological assays focused on the measurement of L2-specific antibody responses.

More than a decade since the first licensure of a L1-based HPV vaccine, many of the initial challenges remained. The need to investigate L2-based vaccine candidates in clinical trials has become increasingly urgent as an alternative approach to overcome inequalities related to the implementation of current HPV vaccination programs worldwide, lack of protection against all oncogenic and possibly oncogenic mucosal HPV types, or against morbidity and treatment costs associated with benign HPV diseases beyond genital wart. The serological assays developed and evaluated in this work represent valuable laboratory approaches to support the clinical investigation of novel HPV vaccines targeting the minor capsid protein L2.

## Data availability statement

The original contributions presented in the study are included in the article/**Supplementary Material**. Further inquiries can be directed to the corresponding author.

## Ethics statement

The study involving humans was reviewed and approved by the Ethics committee University Heidelberg, and a human serum was obtained following a written informed consent (ethics votum S-702/2021). The animal studies conducted at DKFZ were approved by the Regierungspräesidium Karlsruhe, Germany (permit numbers described at M&M). The animal study conducted at Charles River Laboratories was approved by French authorities (project authorization number described at M&M). All animal studies were conducted in accordance with the local legislation and institutional requirements. 

## Author contributions

FCM: Conceptualization, Formal Analysis, Investigation, Methodology, Project administration, Validation, Visualization, Writing – original draft, Writing – review & editing. KP: Formal Analysis, Investigation, Methodology, Writing – review & editing. PS: Formal Analysis, Investigation, Methodology, Validation, Writing – review & editing. MM: Conceptualization, Funding acquisition, Investigation, Project administration, Resources, Supervision, Visualization, Writing – original draft, Writing – review & editing, Methodology.
