# Supplementary material for: Advances on two serological assays for human papillomavirus provide insights on the reactivity of antibodies against a cross-neutralization epitope of the minor capsid protein L2
Source: Front Immunol. 2023 Nov 8;14:1272018. doi: 10.3389/fimmu.2023.1272018 (PMC10663238; doi:10.3389/fimmu.2023.1272018)
Supplement: Supplementary file 1 [file DataSheet_1.docx]

**Advances on two serological assays for human papillomavirus provide insights on the reactivity of antibodies against a cross-neutralization epitope of the minor capsid protein L2**

**Filipe Colaco Mariz^1*^, Kerstin Putzker^2^, Peter Sehr^2^, Martin Müller ^1^**

^1^Tumorvirus-Specific Vaccination Strategies (F035), Deutsches Krebsforschungszentrum (DKFZ), Heidelberg, Germany

^2^EMBL-DKFZ Chemical Biology Core Facility, European Molecular Biology Laboratory, Heidelberg, Germany

*** Correspondence:**Filipe Colaco Mariz
f.mariz@dkfz.de

**Keywords: Human papillomavirus, L2, neutralization assay, ELISA, vaccine, cross-neutralizing antibodies.**

**
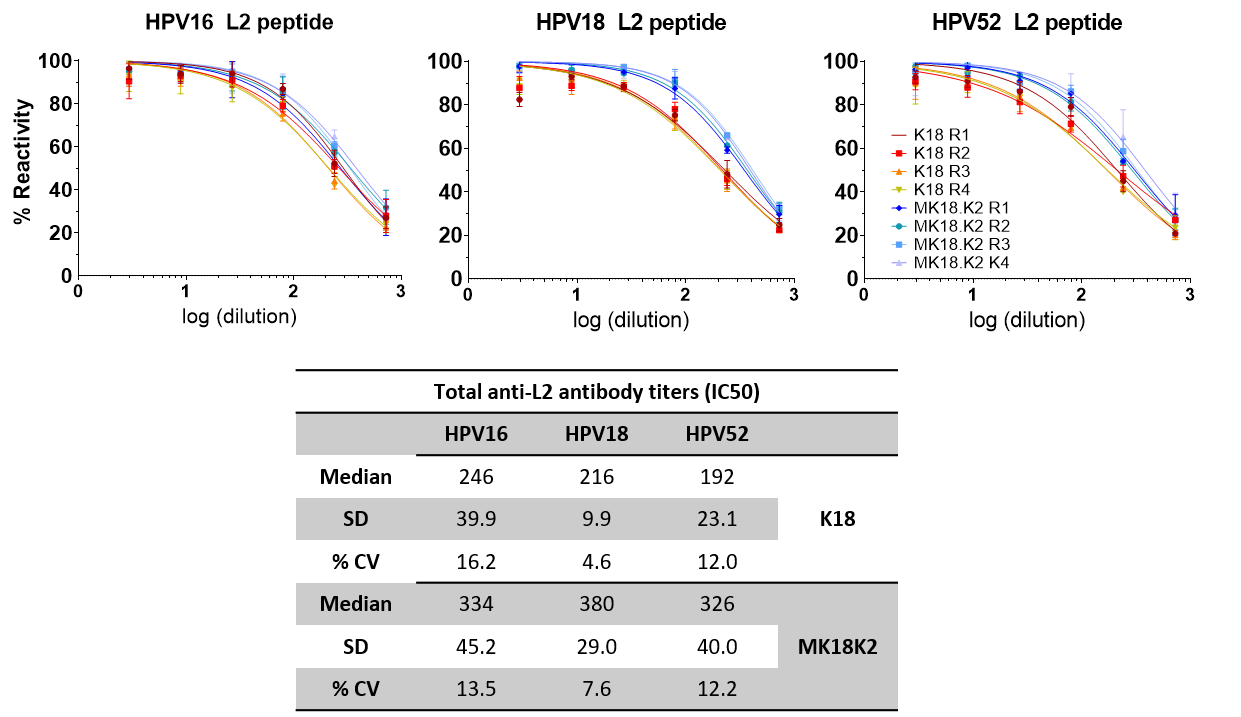
 Supplementary figure 1. Intra-assay variability determined for the L2-peptide ELISA.** The reactivity (%) of two anti-L2 neutralizing monoclonal antibodies (K18 and MK18K2) against HPV16, HPV18 and HPV52 L2 peptides was evaluated via analyses of quadruplicates (R1-4) of each antibody sample. Based on that, IC50 values with corresponding median values, standard deviation (SD) and coefficient of variation (%CV) were calculated for each antibody sample.

**
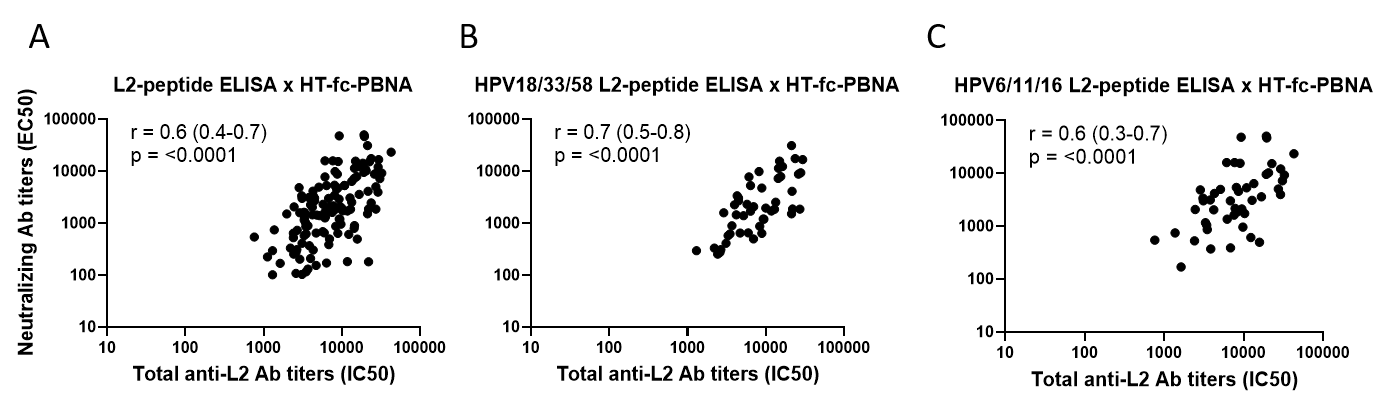
 Supplementary figure 2. Correlation of neutralizing (HT-fc-PBNA) and total antibody titers to the L2 protein according to the peptide oxidation state.** Neutralizing antibody titers measured at days 66 and 79 by HT-fc-PBNA were correlated to the corresponding total anti-L2 antibody titers determined by L2-peptide ELISA for all HPV types **(A)**, and for HPV types to which higher **(B)** or lower peptide oxidation states were measured **(C).** r – Spearman rank correlation coefficients with 95% confidence intervals in parenthesis. P-values determined by the nonparametric Mann–Whitney test.
