# Supplementary material for: Advances on two serological assays for human papillomavirus provide insights on the reactivity of antibodies against a cross-neutralization epitope of the minor capsid protein L2
Source: Front Immunol. 2023 Nov 8;14:1272018. doi: 10.3389/fimmu.2023.1272018 (PMC10663238; doi:10.3389/fimmu.2023.1272018)
Supplement: Supplementary file 2 [file DataSheet_2.docx]

**Advances on two serological assays for human papillomavirus provide insights on the reactivity of antibodies against a cross-neutralization epitope of the minor capsid protein L2**

**Filipe Colaco Mariz^1*^, Kerstin Putzker^2^, Peter Sehr^2^, Martin Müller ^1^**

^1^Tumorvirus-Specific Vaccination Strategies (F035), Deutsches Krebsforschungszentrum (DKFZ), Heidelberg, Germany

^2^EMBL-DKFZ Chemical Biology Core Facility, European Molecular Biology Laboratory, Heidelberg, Germany

*** Correspondence:**Filipe Colaco Mariz
f.mariz@dkfz.de

**Keywords: Human papillomavirus, L2, neutralization assay, ELISA, vaccine, cross-neutralizing antibodies.**

**Supplementary table 1**. Neutralizing antibody titers against the L2 protein of eight mucosal low and high-risk HPV types measured in guinea pig sera and monoclonal antibody preparations.

| Assays | Mean nAb titers (standard error of mean, SEM)^1^ | | | | | | | |
| --- | --- | --- | --- | --- | --- | --- | --- | --- |
|  | **HPV6** | **HPV11** | **HPV16** | **HPV18** | **HPV31** | **HPV33** | **HPV52** | **HPV58** |
| HT-PBNA (EC50) | - | 530 (230) | 47,830 (8,320) | 4,010 (740) | 180 (90) | 1,400 (500) | 80 (30) | 5,120 (1,420) |
| HT-fc-PBNA (EC50) | 13,260 (3,280) | 28,350 (7,190) | 68,720 (10,650) | 13,720 (3,210) | 2,840 (1,980) | 4,850 (1,060) | 510 (230) | 6,480 (1,390) |

^1^Mean antibody titers and corresponding standard error of the mean (SEM) for each HPV type were calculated for groups of eight immune-sera and the two monoclonal antibodies K8 and K18. These titers are also presented in Figure 4 by horizontal bars.
